# Paraneoplastic Immune Thrombocytopenia in Estrogen Receptor (ER)-Positive/Human Epidermal Growth Factor Receptor 2 (HER2)-Positive Advanced Breast Cancer: Clinical Implications and Therapeutic Strategies

**DOI:** 10.7759/cureus.79811

**Published:** 2025-02-28

**Authors:** Rita Antunes Santos, Ana R Coelho, Gonçalo Cunha, Alda Tavares

**Affiliations:** 1 Medical Oncology Department, Instituto Português de Oncologia de Coimbra Francisco Gentil, Coimbra, PRT

**Keywords:** anti-her2 therapy, bone metastasis, breast cancer recurrence, endocrine therapy, thrombocytopenia

## Abstract

This case report describes the clinical course, diagnosis, and management of a premenopausal woman with a history of estrogen receptor (ER)-positive and human epidermal growth factor receptor 2 (HER2)-positive early breast cancer (EBC) who developed severe thrombocytopenia. After six years of the diagnosis of EBC (treated with curative intent), she presented with back pain, significant bruising, and menorrhagia. During the work-up, laboratory tests revealed severe thrombocytopenia, and imaging studies, including computed tomography scan and bone scintigraphy, demonstrated extensive bone lesions and adenopathies. A multidisciplinary approach was crucial in addressing her complex condition, considering potential diagnoses such as immune thrombocytopenia secondary to paraneoplastic syndrome versus hematologic malignancies. Given her history and clinical presentation, the most likely diagnosis was a recurrence of breast cancer with extensive bone metastasis. Despite challenges, including limited biopsy options due to thrombocytopenia, targeted anti-HER2 therapy and endocrine therapy (ET) led to significant clinical improvement.

## Introduction

Breast cancer is the most frequently diagnosed malignant neoplasm and the leading cause of cancer-related death among women worldwide [1]. Approximately 20% of breast cancers overexpress human epidermal growth factor receptor 2 (HER2), as determined by either 3+ staining by immunohistochemistry for the HER2 protein or evidence of HER2 gene amplification by fluorescence in situ hybridization. Overexpression of this receptor is associated with an increased risk of disease recurrence and an overall worse prognosis [2-4]. Without HER2-targeted therapy, 30% to 50% of patients relapse within 10 years [5]. In the case of a diagnosis of HER2-positive metastatic breast cancer, the standard of care is chemotherapy and dual HER2 blockade [6].

Thrombocytopenia is prevalent among cancer patients, primarily due to bone marrow suppression from chemotherapy or radiation therapy. Other less frequent causes include bone marrow infiltration by the cancer itself, drug-induced immune thrombocytopenia, and disseminated intravascular coagulation. Paraneoplastic autoimmune thrombocytopenia (ITP) is a rare cause of thrombocytopenia, requiring a high degree of suspicion, and it has been described most commonly in patients with lung and breast cancer [7,8].

This case report highlights the clinical challenges and therapeutic strategies involved in managing paraneoplastic ITP in a patient with recurrent breast cancer.

## Case presentation

A premenopausal woman in her 40s, with a relevant medical history of obesity, dyslipidemia, and asymptomatic mild pulmonary hypertension, without alteration of biventricular function. Chronically medicated with a statin. There was no family history of oncological disease.

In her 30s, she was diagnosed with invasive ductal carcinoma of the left breast, grade 2, estrogen receptor (ER)-positive/HER2-positive, clinically classified as T2 N1 M0. She underwent neoadjuvant chemotherapy with a regimen of docetaxel (75 mg/m^2^) and cyclophosphamide (600 mg/m^2^) for six cycles, with 21-day intervals, combined with anti-HER2 therapy with trastuzumab (8 mg/kg loading dose, followed by 6 mg/kg every 21 days). This was followed by breast surgery (modified radical mastectomy) with complete axillary lymph node dissection, resulting in a non-complete pathological response - ypT1a(m) ypN1a Lv0 R0. She received locoregional adjuvant radiotherapy due to axillary involvement and continued anti-HER2 therapy for one year. Adjuvant endocrine therapy (ET) with tamoxifen (20 mg daily), associated with ovarian function suppression (OFS), was initiated and continued for five years. Considering the age at diagnosis and the stage of the disease, the extension of ET up to 10 years was considered. However, it was discontinued due to the development of steatohepatitis, confirmed by biopsy and associated with elevated serum aminotransferase levels. Despite this, there was no improvement in parameters after discontinuing tamoxifen, which may be related to the previously described comorbidities.

One year after discontinuing ET plus OFS, the patient presented at the oncology follow-up consultation with a two-month history of back pain, without associated neurological dysfunction, but difficult to control despite analgesia with weak opioids and non-opioids. Nevertheless, she presented with a good performance status. Additionally, she reported heavy menorrhagia and spontaneous bruising scattered across the abdomen and lower limbs, with the same duration. She had no fever or focal signs of infection. Physical examination revealed bilateral cervical adenopathies, supraclavicular adenopathies with the largest conglomerate on the right measuring 40x30 mm, and right pericentrimetric axillary adenopathies, adherent and non-painful. There were no palpable changes in the right breast or left chest wall. There was no hepatomegaly or splenomegaly.

Laboratory findings revealed microcytic anemia, newly developed thrombocytopenia, and elevated levels of alkaline phosphatase and lactate dehydrogenase (LDH), along with increased cancer antigen 15-3 (CA 15-3) and carcinoembryonic antigen (CEA) (Table 1). The hepatic panel showed elevated aminotransferases, which had been previously known and stable, without hyperbilirubinemia (Table 1). There were no abnormalities in coagulation studies or renal function.

**Table 1:** Summary of the patient's laboratory findings. The table highlights key hematological and biochemical abnormalities observed during the course of the disease. Notably, anemia and severe thrombocytopenia were prominent, indicating a complex hematological profile. ULN: Upper limit of normal; LDH: Lactate dehydrogenase; CA 15-3: Cancer antigen 15-3: CEA: Carcinoembryonic antigen

Parameter	Value at Admission	Threshold Recorded Value	Reference Range
Hemoglobin (g/dL)	10.2	6.7	12-16
Platelets (x10³/µL)	62	6	150-400
Lymphocytes (x10³/µL)	15.5	-	1.0-4.8
Alkaline phosphatase (U/L)	4x ULN	-	40-150
LDH (U/L)	3x ULN	-	135-225
CA 15-3 (U/mL)	753	8259	<30
CEA (ng/mL)	105	268	<5

**Figure 1:**
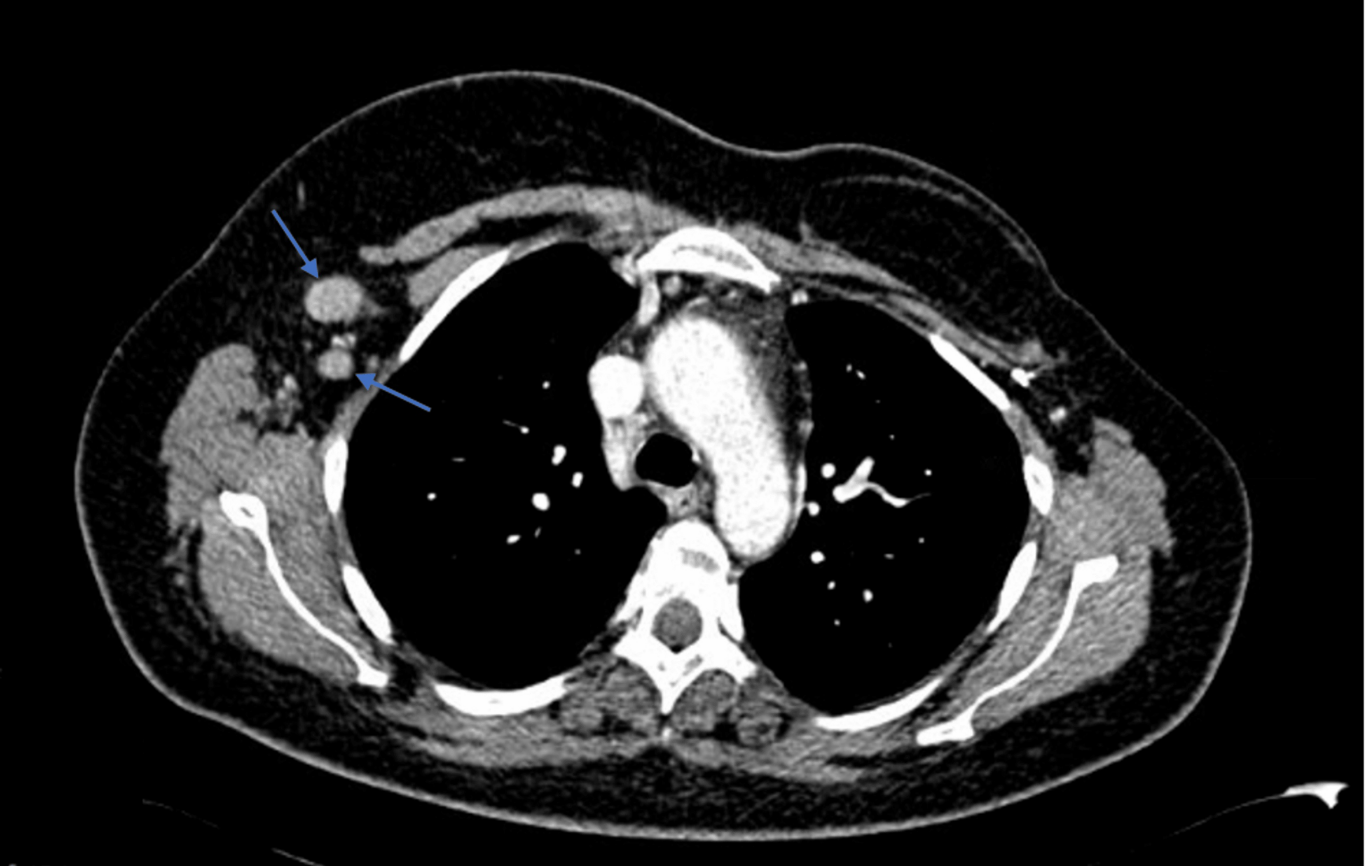
Initial CT showing adenopathies in the right axillary region (arrows). CT: Computed tomography

The patient was admitted for symptomatic control and further investigation. An axillary ultrasound revealed multiple lymph nodes on the right, the largest measuring 33x18 mm, warranting histological characterization. A computed tomography scan of the cervical, thoracic, abdominal, and pelvic regions revealed multiple enlarged lymph nodes in both lateral cervical chains, supraclavicular fossae, and the right axillary region (Figure 1), as well as extensive structural bone changes involving almost the entire skeleton, consistent with massive metastasis; there were no hepatic or splenic abnormalities.

Serial laboratory evaluations conducted over two weeks revealed worsening hematological abnormalities, notably microcytic anemia and thrombocytopenia, which required frequent red blood cell and platelet transfusions (Table 1). Additionally, lymphocytosis was noted. The peripheral blood smear (PBS) indicated mature lymphocytosis with pleomorphism and the presence of "smudge" cells, with no morphologic platelet abnormalities or alterations in the erythroid lineage. The sedimentation rate, C-reactive protein, and ferritin levels were elevated. The antiplatelet antibody tested positive. Serum protein electrophoresis showed no monoclonal peak, while antinuclear antibodies and the Coombs test were negative, and haptoglobin levels were normal. Serologies for human immunodeficiency virus, hepatitis B and C, Epstein-Barr virus, and cytomegalovirus were all negative. Flow cytometry and immunophenotyping could not be performed due to extensive cell lysis. At this stage, lymph node and bone marrow biopsies could not be conducted due to limiting thrombocytopenia.

During the investigation, several diagnostic hypotheses were considered, including the possibility of a lymphoproliferative disease - specifically chronic lymphocytic leukemia - due to the presence of adenopathies, lymphocytosis, and suggestive findings on the PBS, such as "smudge" cells.

Other issues discussed included microcytic anemia and symptomatic thrombocytopenia, and how they might relate to the overall clinical picture. Severe thrombocytopenia limited the diagnostic workup, making biopsies unsafe to perform. For this reason, hematologists were involved early on to provide the best discussion of this case. Given the patient's previous history of invasive breast carcinoma and the rise in tumor markers (CEA and CA 15-3), the most likely diagnostic hypothesis was a recurrence of breast cancer with extensive lymph node metastasis (cervical, supraclavicular, and axillary) and diffuse bone involvement, with the hematological abnormalities considered secondary to a progressing metastatic process.

Thus, lymphocytosis was thought to be associated with an inflammatory neoplastic context. The presence of anti-platelet antibodies suggested ITP in a paraneoplastic context after excluding other secondary causes such as viral infections or autoimmune diseases. Microcytic anemia was considered multifactorial in etiology due to its inflammatory state and significant blood loss in the form of menorrhagia, exacerbated by thrombocytopenia.

Multiple therapeutic issues were addressed during the hospitalization. In addition to the early involvement of hematologists, palliative care was also necessary to support symptomatic control. The back pain was initially difficult to manage, requiring progressively increasing doses of strong opioids, in combination with non-opioids and adjuvants. Vaginal blood loss worsened throughout the hospitalization, necessitating the administration of antifibrinolytics for better control. Due to frequent hemorrhagic episodes, hemoglobin levels were severely affected, requiring weekly transfusions.

Regarding thrombocytopenia, it was initially managed with recurrent platelet transfusions. In a second approach, after knowing the presence of antiplatelet antibodies, high-dose corticosteroid therapy (prednisolone 1 mg/kg/day) was initiated but had no impact on increasing platelet counts. Due to the high risk of hemorrhage, the patient had to remain on strict bed rest for an extended period. An advance directive was addressed and issued.

Concerning the probable recurrence of breast cancer, hematological abnormalities limited the selection of an optimal systemic palliative therapy, particularly regarding the introduction of chemotherapy. Therefore, after a multidisciplinary oncology team discussion and considering the patient's good performance status, it was decided to initiate anti-HER2 therapy (trastuzumab, 8 mg/kg loading dose, followed by 6 mg/kg every 21 days, intravenous infusion), combined with OFS (goserelin, 3.6 mg every 28 days, subcutaneous injection) and ET (letrozole, 2.5 mg/day, oral, starting after six weeks of first administration of goserelin).

**Figure 2:**
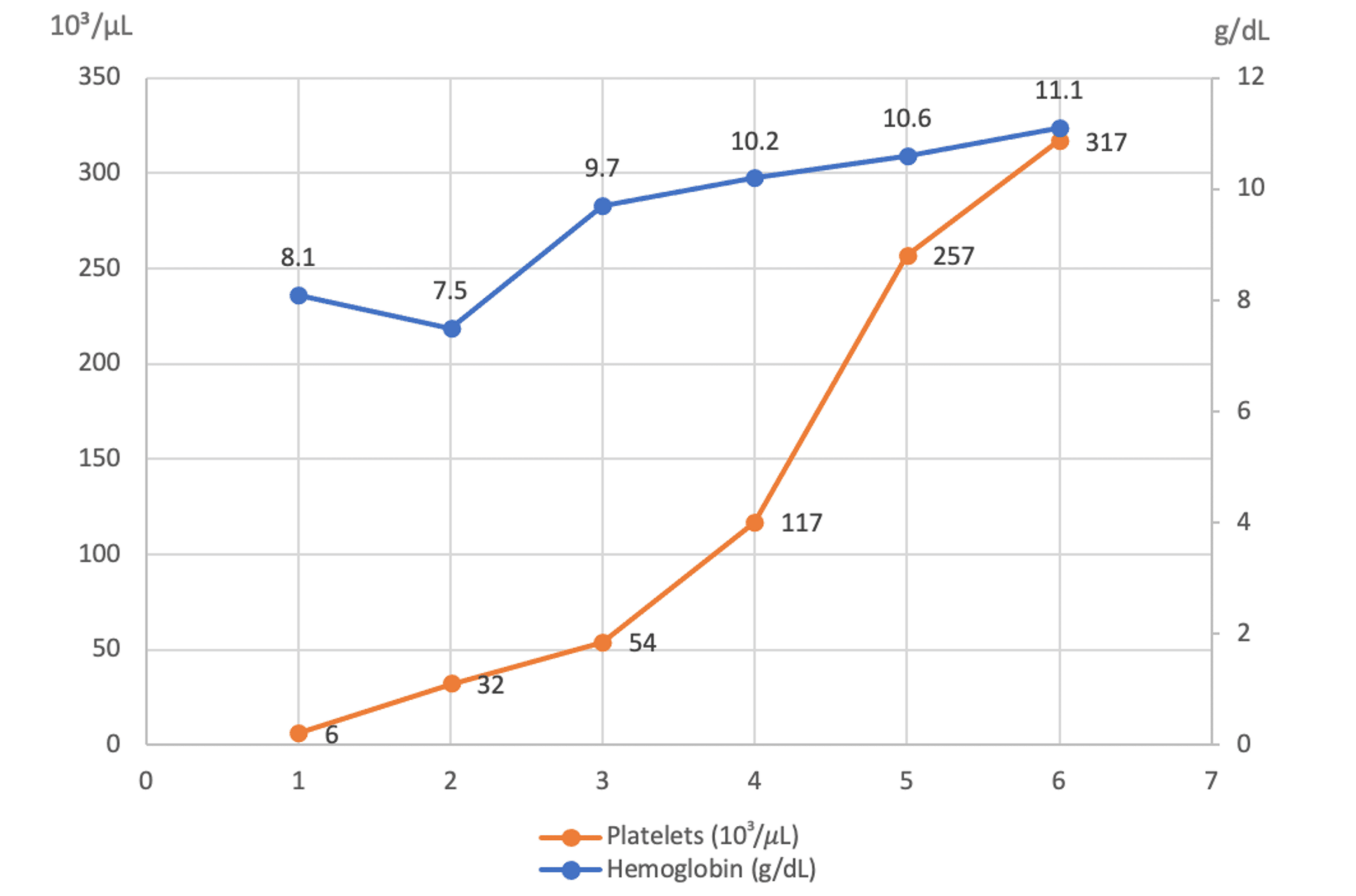
Hematologic recovery after the initiation of targeted anti-HER2 therapy (trastuzumab, cycles of 21 days), combined with goserelin and letrozole. HER2: Human epidermal growth factor receptor 2

**Figure 3:**
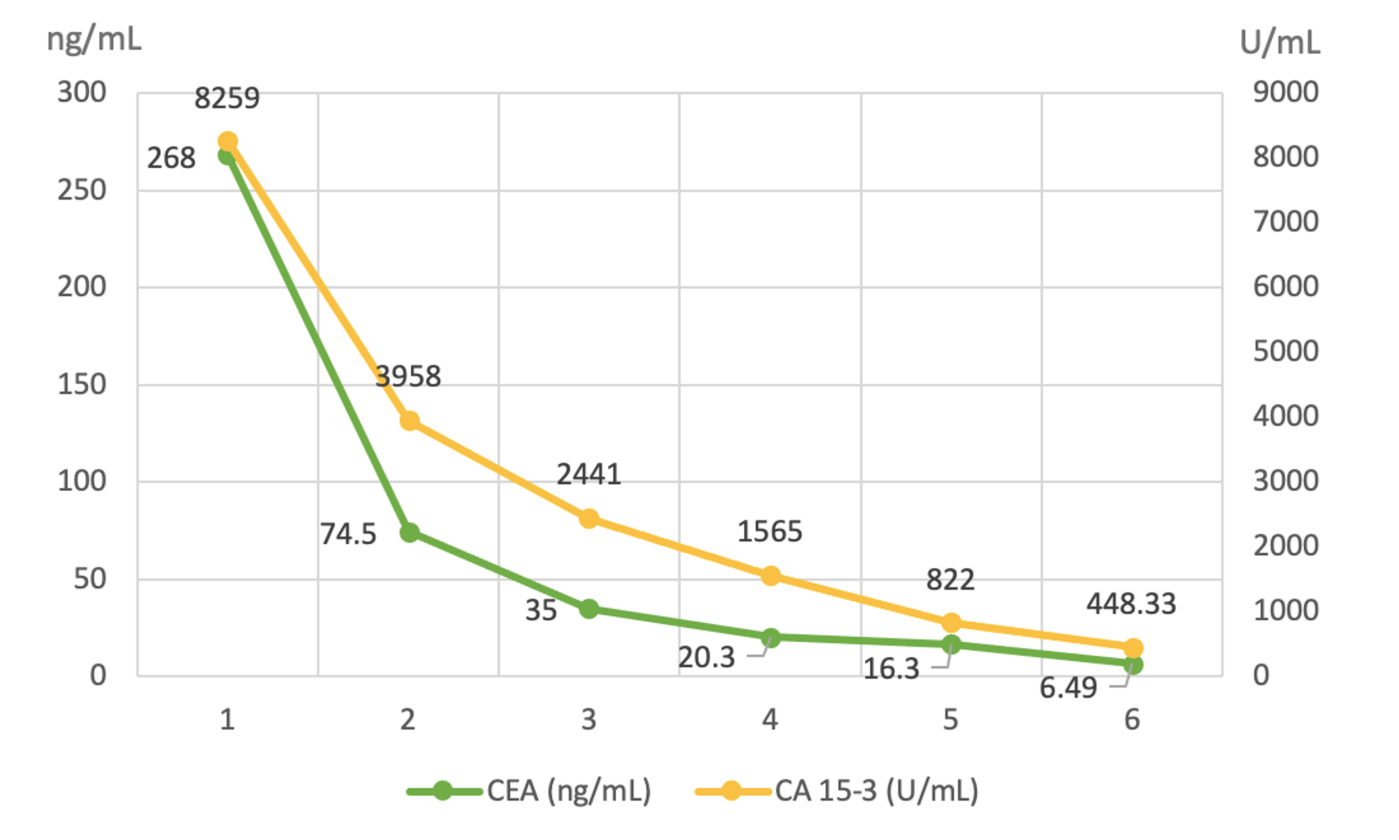
Decrease in tumor markers after the initiation of targeted anti-HER2 therapy (trastuzumab), combined with goserelin and letrozole. HER2: Human epidermal growth factor receptor 2; CA 15-3: Cancer antigen 15-3: CEA: Carcinoembryonic antigen

**Figure 4:**
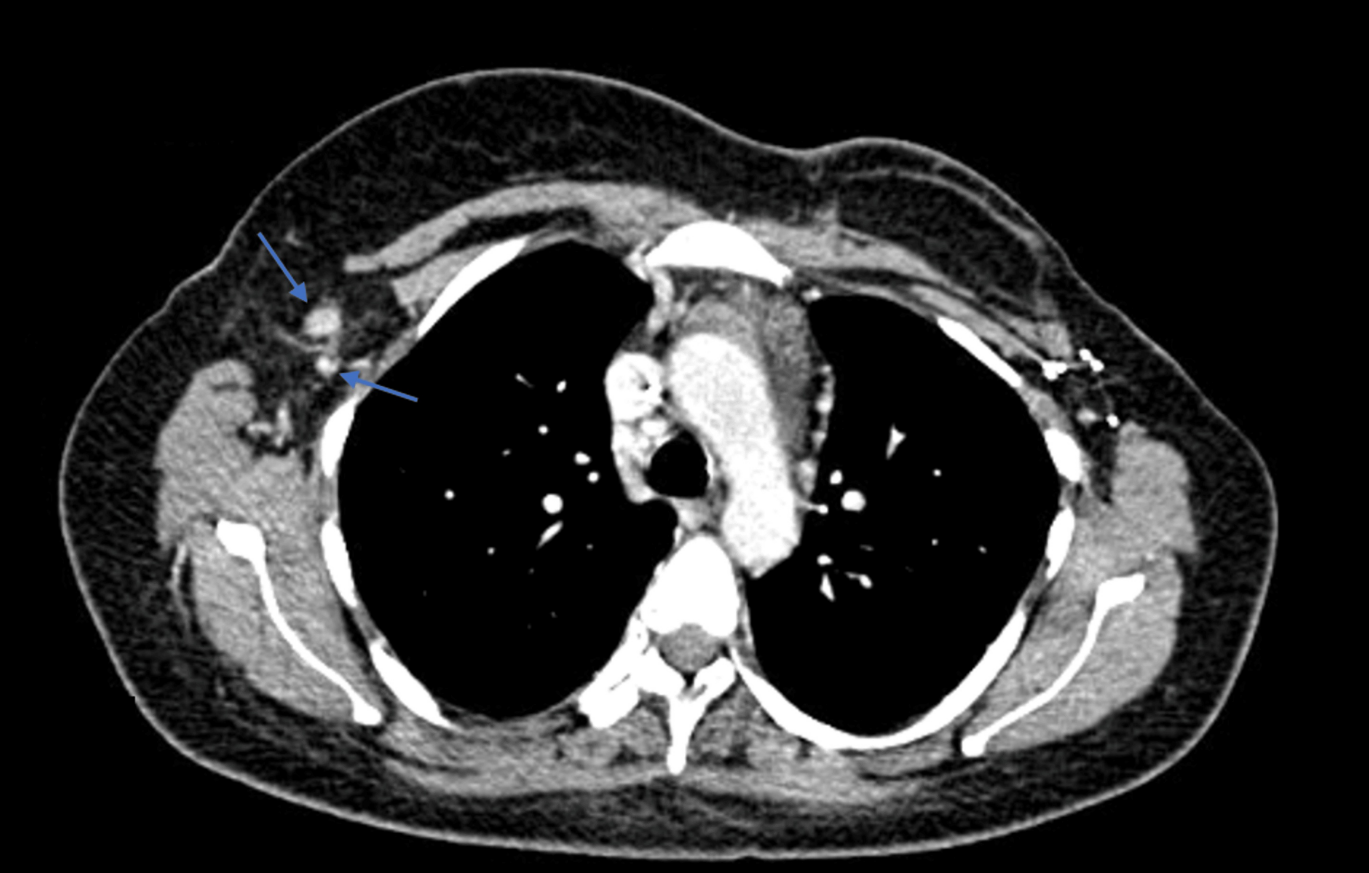
Reduction of the adenopathies after the initiation of targeted anti-HER2 therapy (trastuzumab), combined with goserelin and letrozole (arrows). HER2: Human epidermal growth factor receptor 2

After the initiation of trastuzumab, combined with goserelin (and posterior letrozole), the patient exhibited very favorable clinical and laboratory progress. This included a reduction in the size of cervical, supraclavicular, and right axillary lymph nodes, better pain control, as well as less blood loss and ecchymosis, progressive hematologic recovery (Figure 2), and a decrease in tumor markers (Figure 3). She completed six cycles of trastuzumab, plus goserelin and letrozole, with recovery from thrombocytopenia and a reduction in lymph node sizes (Figure 4). A biopsy of the right axillary lymph node confirmed metastasis of carcinoma with morphology and immunophenotype consistent with the primary breast cancer, ER-positive/HER2-positive. The patient was also able to resume her daily activities and return to work, significantly improving her quality of life.

She continued with optimization of systemic therapy, initiating a taxane (docetaxel) combined with dual HER2 blockade (trastuzumab and pertuzumab), completing six cycles, followed by maintenance of dual blockade, along with ET and OFS.

After one year from the start of the first-line palliative therapy, imaging showed disease progression at the nodal level, with the appearance of mediastinal adenopathies; however, there was no hematologic deterioration. A second line of anti-HER2 therapy was initiated, specifically trastuzumab emtansine (T-DM1), 3.6 mg/kg every 21 days, intravenous infusion. After eight months, she developed severe headaches, secondary to leptomeningeal carcinomatosis (documented in magnetic resonance imaging). She died after two years of the diagnosis of advanced breast cancer.

## Discussion

According to the American Society of Hematology guidelines, ITP is an acquired autoimmune disorder, with an estimated incidence of two to five per 100,000 persons in the general population. ITP is a heterogeneous disorder and remains a diagnosis of exclusion of other causes of thrombocytopenia [9]. It can be an isolated primary condition or secondary to other conditions, such as lymphoproliferative disorders, which comprise up to 30% of secondary ITP cases [9,10].

Paraneoplastic syndromes are commonly associated with malignancy and can occasionally present as immune-mediated hematological syndromes [8,11]. Paraneoplastic secondary ITP in solid tumors is less well known but has been documented. Several case series in the literature report the diagnosis of ITP in breast cancer patients, some of which present as the initial indication of cancer recurrence [7,11].

The pathophysiological mechanism of paraneoplastic ITP is not fully understood, which could have significant implications for its diagnosis and treatment. Low platelet counts can potentially delay adequate cancer investigation, as demonstrated in this clinical case. This underscores the critical need for a multidisciplinary approach in assessing complex differential diagnoses and highlights the importance of a comprehensive understanding of the natural progression of oncological diseases.

Additionally, this case emphasizes the complexities involved in therapeutic decision-making, particularly in balancing the risks of potential toxicity against the anticipated clinical benefits. Evidence suggests that in cases of ITP secondary to solid tumors, aggressive treatment of the primary malignancy can effectively mitigate the triggers of autoimmunity, leading to significant clinical improvement [12]. In this case, the risk of fatal bleeding was high. Standard of care at the first line was not possible. After discussion and a shared decision with the patient, trastuzumab and goserelin (and posterior letrozole) were started, considering the hematological toxicity profile practically null with these drugs. Expertise in the management of oncologic drugs, especially targeted therapies such as anti-HER2 agents and ET, is crucial and can potentially improve patient outcomes. 

## Conclusions

Paraneoplastic syndromes, such as ITP, are critical considerations in patients with a history of malignancies, including breast cancer. These syndromes may occasionally serve as an early indicator of cancer recurrence, highlighting the need for vigilant clinical assessment. Additionally, hematologic abnormalities, particularly thrombocytopenia, can significantly impact cancer treatment strategies by limiting the feasibility of chemotherapy, necessitating the exploration of alternative therapeutic approaches. This underscores the importance of personalized medicine, where treatment plans are tailored to the individual patient's clinical profile to optimize therapeutic outcomes. A comprehensive understanding of these factors is essential for improving patient management and ensuring the best possible prognosis.

## References

[1] Tevaarwerk AJ, Gray RJ, Schneider BP (2013). Survival in patients with metastatic recurrent breast cancer after adjuvant chemotherapy: little evidence of improvement over the past 30 years. Cancer.

[2] Cobleigh MA, Vogel CL, Tripathy D (1999). Multinational study of the efficacy and safety of humanized anti-HER2 monoclonal antibody in women who have HER2-overexpressing metastatic breast cancer that has progressed after chemotherapy for metastatic disease. J Clin Oncol.

[3] Slamon DJ, Leyland-Jones B, Shak S (2001). Use of chemotherapy plus a monoclonal antibody against HER2 for metastatic breast cancer that overexpresses HER2. N Engl J Med.

[4] Mass RD, Press MF, Anderson S (2005). Evaluation of clinical outcomes according to HER2 detection by fluorescence in situ hybridization in women with metastatic breast cancer treated with trastuzumab. Clin Breast Cancer.

[5] O'Shaughnessy J, Gradishar W, O'Regan R, Gadi V (2023). Risk of recurrence in patients with HER2+ early-stage breast cancer: literature analysis of patient and disease characteristics. Clin Breast Cancer.

[6] Swain SM, Baselga J, Kim SB (2015). Pertuzumab, trastuzumab, and docetaxel in HER2-positive metastatic breast cancer. N Engl J Med.

[7] Jo T, Okamoto Y, Tsukamoto T (2013). 27 cases of paraneoplastic autoimmune thrombocytopenia in solid tumors. Ann Oncol.

[8] Krauth MT, Puthenparambil J, Lechner K (2012). Paraneoplastic autoimmune thrombocytopenia in solid tumors. Crit Rev Oncol Hematol.

[9] Neunert C, Terrell DR, Arnold DM (2019). American Society of Hematology 2019 guidelines for immune thrombocytopenia. Blood Adv.

[10] Visco C, Rodeghiero F (2009). Immune thrombocytopenia in lymphoproliferative disorders. Hematol Oncol Clin North Am.

[11] Puthenparambil J, Lechner K, Kornek G (2010). Autoimmune hemolytic anemia as a paraneoplastic phenomenon in solid tumors: A critical analysis of 52 cases reported in the literature. Wien Klin Wochenschr.

[12] Ghanavat M, Ebrahimi M, Rafieemehr H, Maniati M, Behzad MM, Shahrabi S (2019). Thrombocytopenia in solid tumors: prognostic significance. Oncol Rev.

